# Completely stopping microwaves with extremely enhanced magnetic fields

**DOI:** 10.1038/s41598-018-33956-0

**Published:** 2018-10-25

**Authors:** Qian Shen, Lujun Hong, Xiaohua Deng, Linfang Shen

**Affiliations:** 1 Institute of Space Science and Technology, Nanchang University, Nanchang, 330031 China; 2 Department of Information Engineering, Nanchang University, Nanchang, 330031 China

## Abstract

A microwave one-way waveguide of three-dimensional configuration is proposed and investigated theoretically. In this waveguide there exists a complete one-way propagation band, where the mode propagates only in one direction and can be immune to backscattering. By terminating the one-way waveguide with metal slab, one-way propagating waves in this waveguide system can be stopped at the terminal end without any backscattering. Meanwhile, a hotspot with extremely enhanced magnetic-field amplitude is generated in this 3D waveguide system. For an incident microwave pulse, the trapped wave packet can be compressed to deep subwavelength scale besides the magnetic field enhancement. Moreover, the magnetic field enhancement of trapped waves can be further largely increased by tapering laterally the waveguide system. The approach for trapping microwaves has promising applications in magnetic sensing and magnetic non-linearity.

## Introduction

Completely stopping light or electromagnetic (EM) radiation in all-optical systems is not only of great interest in fundamental science, but also has a variety of prospective applications such as light harvesting^1,2^, sensing^3^, and enhanced non-linearity^4^. It was reported that surface plasmon polaritons (SPPs) can be stopped in a tapered plasmonic waveguide, where the group velocity of SPPs asymptotically reduces to zero^1^. The stopped SPPs generate a hotspot with giant local fields. Various tapered waveguides which were made of metamaterials were proposed later^5–7^, and the phenomenon of trapped rainbow was also reported^6,7^, in which incident light or EM waves with different frequencies stop at correspondingly different locations. The trapped rainbow is particularly attractive for the applications in optical data storage. However, some researchers recently pointed out that incident EM waves in these tapered waveguides with metamaterials were totally reflected instead of being trapped^8^. There were also other approaches for stopping light which are based on dynamic processes, and with these methods, light can be coherently stored in coupled resonator optical waveguide systems^9–11^. Recently, it was further reported that by using one-way surface magnetoplamons (SMPs), terahertz radiations can be completely stopped in a forcible and rapid way^12,13^. Such method of stopping EM waves seems to be meaningful in practice, because relevant optical systems often include dispersive materials (e.g., plasmonic materials) that are inherently lossy. Moreover, this method is robust against imperfections in the system.

One-way SMPs can be sustained by plasmonic materials under applied dc magnetic fields, which break the time-reversal symmetry of the systems^14,15^. Due to the nonreciprocity, the asymptotic frequency of SMPs differs in the forward and backward directions, thus SMPs are allowed to unidirectionally propagate in the frequency interval between the two different asymptotic frequencies. It has been shown that one-way SMPs can be immune to backscattering at imperfections or bends, because of the absence of back-propagating modes in the system. By using semiconductors, which are gyro-electrically anisotropic under magnetization, one-way SMPs at terahertz frequencies can be realized with external magnetic field of normal intensity^15–17^. When such one-way SMPs are blocked, they can be completely trapped without any backscattering. Meanwhile, hotspots with extremely enhanced electric fields are generated^12,13,18^, which have promising applications in terahertz sensing and enhanced non-linearity. By using yttrium-iron-garnet (YIG) material, which is gyro-magnetic under magnetization, one-way SMPs can also be realized at microwave frequencies^19,20^. Moreover, one-way regular mode, which is guided by the mechanism of total internal reflection, has also been reported at microwave frequencies^21^. Recently, Liu proposed an approach to achieve trapped rainbow at microwave frequencies based on one-way SMPs^22^. In the Liu’s system, a linearly tapered external magnetic field was used. For such tapered magnetic field, it seems that a steady electric current is required to be present in the YIG. In addition, based on the zero group velocity of SMPs, the trapping behaviour would be seriously affectted by the material loss in practice.

All one-way SMPs at terahertz or microwave frequencies reported previously are spatially two-dimensional (2D), i.e., their fields as well as the related systems are uniform and extend infinitely in the lateral direction. This is also the situation for trapped hotspots based on one-way SMPs. In this paper, a realistic one-way waveguide, which is a three-dimensional (3D) structure constructed by using YIG, is proposed and investigated theoretically. We will show that this waveguide can sustain robust one-way mode at microwave frequencies. Furthermore, we propose a simple and effective approach to trap microwaves in a 3D system by using the one-way waveguide. In this system, only a uniform dc magnetic field is needed. Compared to Liu’s approach^22^, wave trapping would be available for our approach even in the cases with serious material losses, and the related magnetic-field enhancement would be weakly affected by the material loss. On the other hand, different from the effect of wave trapping in refs^12,13^, wave trapping in our system would lead to extreme magnetic-field enhancement, instead of the electric-field enhancement. All above properties for the proposed 3D waveguide system will be numerically demonstrated in detail. Moreover, we will show that by tapering laterally the 3D system for trapping wave, the field enhancement can be further remarkably increased.

## Realistic Microwave One-Way Waveguides

**Figure 1:**
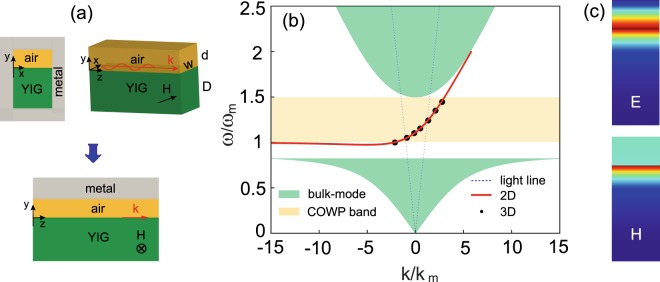
(**a**) Upper left panel, the crossection of the 3D one-way waveguide; upper right panel, the internal structure of the 3D waveguide; lower panel, the 2D system physically equivalent to the 3D waveguide. (**b**) Dispersion relations for the 2D (solid line) and 3D (solid circles) guiding systems. The uppermost and lowest shaded areas represent the zones of bulk modes in the YIG, and the middle rectangular one represents the COWP region for the 2D and 3D systems. (**c**) Modal fields at 5.6 GHz. The parameters of the systems are as follows: *ε*_*m*_ = 15, *ω*_0_ = 0.5 *ω*_*m*_, and *ν* = 0; *d* = 3 mm, *w* = 5 mm, and *D* = 9 mm.

The realistic one-way waveguide we consider is formed by a rectangular metallic waveguide partly filled with YIG (for the region *y* < 0), as illustrated in the upper panels in Fig. 1(a). The waveguide width is denoted by *w* (in the *x* or lateral direction) and the YIG thickness denoted by *D*. The remaining region within the metallic waveguide is filled with air, whose thickness is denoted by *d*. The upper right panel in Fig. 1(a) shows the internal structure of the guiding system. In this system, a uniform dc magnetic field (*H*_0_) is applied in the *x* direction. The magnetized YIG has the relative permittivity *ε*_*m*_ = 15 and the relative permeability ${\overleftrightarrow{\mu }}_{m}$ in the form^20^

1 $${\overleftrightarrow{\mu }}_{m}=[\begin{array}{ccc}1 & 0 & 0\\ 0 & {\mu }_{1} & i{\mu }_{2}\\ 0 & -i{\mu }_{2} & {\mu }_{1}\end{array}],$$

with

2 $${\mu }_{1}=1+\frac{{\omega }_{m}\,({\omega }_{0}-i\nu \omega )}{{({\omega }_{0}-i\nu \omega )}^{2}-{\omega }^{2}},$$

3 $${\mu }_{2}=\frac{\omega {\omega }_{m}}{{({\omega }_{0}-i\nu \omega )}^{2}-{\omega }^{2}},$$

where *ω* is the angular frequency, *ω*_0_ = 2*πγH*_0_ (where *γ* is the gyromagnetic ratio) being the precession angular frequency, *ω*_*m*_ is the characteristic circular frequency of the YIG, and *ν* is the damping coefficient. Here, the metal is assumed to be perfect electric conductor, which is a good approximation for the microwave regime. We suppose that the waveguide width is enough small, i.e., $w\ll {\lambda }_{m}$, where *λ*_*m*_ is the vacuum wavelength for *ω*_*m*_. Thus, in a frequency range around *ω*_*m*_, the waveguide can only support TE modes with uniform electric field pointing in the *x* direction. In the case when the YIG thickness is enough large, the guiding modes in such a 3D system should have almost the same properties as those in a 2D guiding system, which is a YIG-air-metal layered structure shown in the lower panel in Fig. 1(a).

In the band gap of the magnetized YIG, the 2D system in Fig. 1(a) can support not only SMPs but also regular mode, the latter is guided by the mechanism of total internal reflection^21^. Both modes are TE polarized. The dispersion relation of modes, which associates the propagation constant (*k*) with *ω*, can be derived analytically. It has the form

4 $${\alpha }_{d}{\mu }_{{\rm{v}}}+({\alpha }_{m}+\frac{{\mu }_{2}}{{\mu }_{1}}k)\tanh ({\alpha }_{d}d)=0,$$

for SMPs with |*k*| > *k*_0_, where ${\alpha }_{d}=\sqrt{{k}^{2}-{k}_{0}^{2}}$ with *k*_0_ = *ω*/*c* (where c is the light speed in vacuum), and ${\alpha }_{m}=\sqrt{{k}^{2}-{\varepsilon }_{m}{\mu }_{{\rm{v}}}{k}_{0}^{2}}$ with ${\mu }_{{\rm{v}}}={\mu }_{1}-{\mu }_{2}^{2}/{\mu }_{1}$ being the Voigt permeability. The dispersion relation becomes

5 $$p{\mu }_{{\rm{v}}}+({\alpha }_{m}+\frac{{\mu }_{2}}{{\mu }_{1}}k)\tan (pd)=0,$$

for regular mode with |*k*| < *k*_0_, where $p=\sqrt{{k}_{0}^{2}-{k}^{2}}$. Obviously, both types of mode are non-reciprocal, because there exists a linear term with respect to *k* in Eqs (4) and (5). The dispersion bands for SMPs lie outside of the light cone (defined by air), and its left branch (*k* < 0) has an asymptotic frequency of *ω*_*sp*_ = *ω*_0_ + *ω*_*m*_/2, at which *k* → −∞^21^. The dispersion band for the regular mode lies within the light cone, and it links with the SMP bands at points of *ω* = *c*|*k*|, thus forming a complete dispersion band over a wide frequency range.

Figure 1(b) shows the dispersion relation (solid line) for the 2D guiding system. In this paper, we take *ω*_0_ = 0.5*ω*_*m*_ and *ω*_*m*_ = 10*π* × 10^9^ rad/s for the magnetized YIG, and *d* = 0.05*λ*_*m*_ (*λ*_*m*_ = 2*πc*/*ω*_*m*_) for air. As shown in Fig. 1(b), a complete dispersion band occurs, which intersects with the light lines. There exists a piece of complete one-way propagation (COWP) band with positive slope, which is located within the band gap of the YIG. Evidently, the mode on the COWP band can only propagate forward. The COWP band is between *ω*_*sp*_ and *ω*_*cf*_, where *ω*_*cf*_ = *ω*_0_ + *ω*_*m*_, and it corresponds to a frequency range from 5 GHz to 7.5 GHz. The COWP region is indicated by a shaded rectangle in Fig. 1(b). The dispersion relation for the one-way mode in our 3D system should be the same as that for the 2D system. To verify this, we numerically solve for modes in the 3D system with the finite-element method (FEM) (by using the COMSOL Multiphysics). For the 3D system, the waveguide width is *w* = 5 mm (i.e., *w* = *λ*_*m*_/12) and the YIG thickness *D* = 9 mm. The calculated dispersion relation is plotted as solid circles in Fig. 1(b), which agrees well with that for the 2D system. Figure 1(c) shows the field amplitudes of the mode in the 3D system for 5.6 GHz (i.e., *ω* = 1.12 *ω*_*m*_), at which *k* = 0, and as expected, both the *E* and *H* fields are uniform in the *x* direction, and they peak at the interface of the air and YIG, like the fields of one-way SMPs.

**Figure 2:**
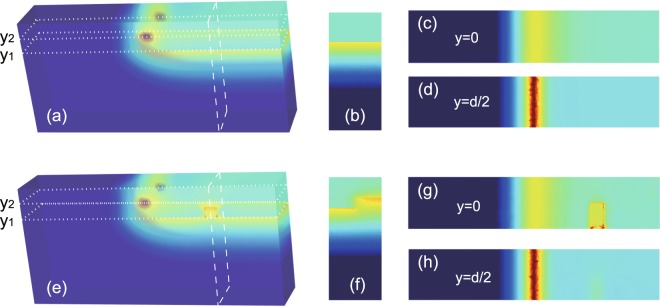
Simulated magnetic field amplitudes for the 3D guiding systems without (**a**) and with (**e**) obstacle. The obstacle in (**e**) is a square YIG column with a side length of 1 mm and it lies on the half of the YIG surface. (**b**) Vertical cutting slice in (**a)** at 30 mm away from the source, (**c**) and (**d**) are the horizontal cutting slices in (**a**) at *y*_1_ = 0 and *y*_2_ = *d*/2, respectively. (**f**–**h**) Three corresponding slices in (**e**). The operation frequency is *f* = 5.6 GHz, and the parameters of the guiding system are the same as in Fig. 1.

To validate the guiding properties of the 3D system, we performed the simulation of wave transmission in it with the FEM. In the simulation, a linear current source of the length *w* was placed at *y* = *d*/2 to excite waves. The operation frequency was set at *f* = 5.6 GHz. Figure 2(a) shows the simulated magnetic field amplitudes. Clearly, the excited wave is only propagating forward as expected. Figure 2(b) corresponds to the field amplitudes on a vertical cutting slice at a (horizontal) distance of 30 mm away from the source, while Fig. 2(c,d) are the field amplitudes on the horizontal cutting slices at *y* = 0 and *y* = *d*/2, respectively. We further examined the robustness of the one-way mode. For this purpose, we introduced an obstacle into the system in Fig. 2(a), and it was placed at a (horizontal) distance of 30 mm from the source. The obstacle is a square YIG column with a side length of 1 mm. It lies on the half of the YIG surface, and its length is equal to *w*/2. The system with the obstacle becomes asymmetric in the *x* direction. The simulated magnetic field amplitudes are plotted in Fig. 2(e–h). Clearly, the one-way propagating wave goes around the obstacle and continues to travel forward. The lateral uniformity of field is broken only in the local region of the obstacle, but is completely recovered behind it. Therefore, the one-way mode can be immune to backscattering in our 3D system.

## Wave Stopping and Magnetic-Field Enhancement

**Figure 3:**
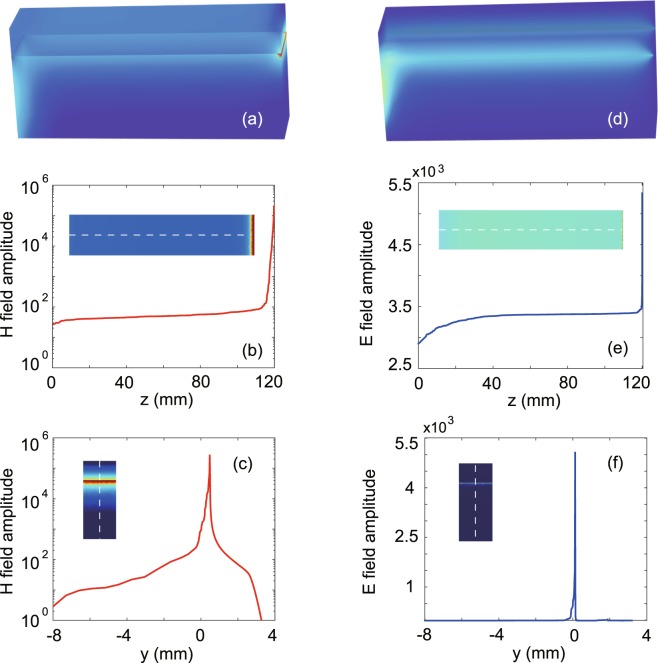
Simulated field amplitudes for the complete trapping of one-way propagating wave. (**a**) *H* amplitudes. (**b**) Distribution of *H* amplitude along the central line of the YIG surface. The inset is a horizontal cutting slice in (**a**) at *y* = 0. (**c**) Distribution of *H* amplitude along the y axis at a distance of 0.02 mm before the terminal end. The inset is a vertical cutting slice in (**a**) at *z* = 119.98 mm, where the center of the hotspot is located. (**d**–**f**) *E* amplitude versions corresponding to (**a**–**c**), respectively. The operation frequency is *f* = 5.6 GHz, *ν* = 10^−3^, and the other parameters are the same as in Fig. 1.

Suppose that the one-way waveguide is terminated by a metal slab in the propagation direction. In this waveguide system, waves traveling forward in it will be blocked when they reach the terminal end. In the COWP range, there exists no backward-propagating mode in the waveguide, therefore the blocked waves must be completely stopped at the terminal end without any backscattering. To verify this, by using the FEM, we simulated wave transmission in the terminated waveguide system with a length of 120 mm. In the simulation, the open waveguide end at *z* = 0 was excited using an input port with a TE_01_ mode, and the frequency was *f* = 5.6 GHz. To obtain a steady solution, we took the loss of YIG into account, and set *ν* = 10^−3^. The simulated magnetic field amplitudes are plotted in Fig. 3(a). Clearly, a hotspot with extremely enhanced magnetic field occurs near the terminal end. Figure 3(b) shows the field distribution along the central line of the YIG surface. The center of the hotspot is found at *z* = 119.98 mm. Figure 3(c) shows the field profile along the *y* axis at *z* = 119.98 mm. The simulated electric field amplitudes are plotted in Fig. 3(d), and no remarkable field enhancement is observed. Figure 3(e,f) are the electric-field profiles similar to Fig. 3(b,c).

As the electric field of the trapped wave points in the x direction, it vanishes at the terminal end which is formed by a metal slab. Consequently, the hotspot generated by trapping wave occurs near the terminal end but peaks at a small distance away from it. The hotspot corresponds to the accumulation of EM energy in the region of subwavelength scale, and for sustaining it, there must exist a large number of electric or “magnetic” charges locally on the YIG surface. These charges are impossible to be electrons, because the electric field is tangential to the interface of YIG and air. However, the magnetic field of the trapped wave has a nonzero component normal to the interface, so “magnetic” charges can be effectively excited and heaped at the YIG-air interface. In the YIG, “magentic” charges also exist with high density within the skin layer, and their density corresponds to the divergence of the magnetic moment. As a result of trapping wave, the magnetic field amplitudes are extremely enhanced, while the electric field amplitudes remain at a usual level. To characterize the field enhancement by trapping wave, we performed the simulation of wave transmission once more, but the length of the one-way waveguide was increased to 360 mm. The simulated magnetic (electric) field amplitude at the propagation distance of 120 mm (on the YIG surface) is used to normalize the maximal magnetic (electric) field amplitude of the hotspot in Fig. 3, and the result ${\bar{H}}_{{\rm{\max }}}$ (${\bar{E}}_{{\rm{\max }}}$) is used to represent the field enhancement. It is found that ${\bar{H}}_{{\rm{\max }}}$ = 1800 and ${\bar{E}}_{{\rm{\max }}}$ = 1.85. Therefore, by completely stopping wave, the magnetic field amplitude is enhanced by 3 orders of magnitude at the hotspot.

**Figure 4:**
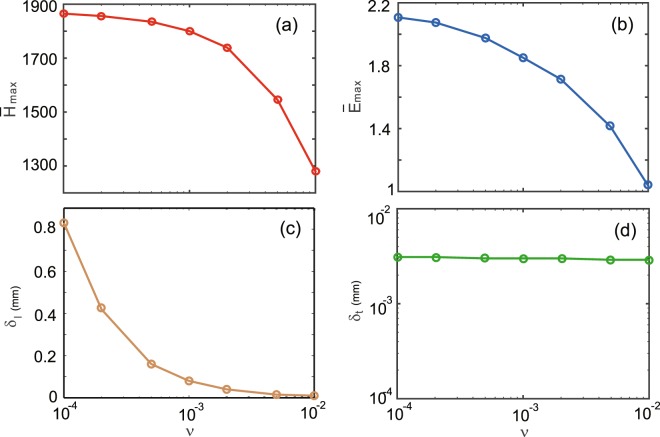
Dependences of ${\bar{H}}_{{\rm{\max }}}$, ${\bar{E}}_{{\rm{\max }}}$, *δ*_*l*_, and *δ*_*t*_ on *ν*. The frequency is *f* = 5.6 GHz, and the other parameters are the same as in Fig. 1.

We further investigate the influence of the YIG loss on the hotspot. To clarify this, several *ν* values are numerically analyzed, and besides the field enhancement, the hotspot sizes are also evaluated. The longitudinal (transverse) size of the hotspot can be defined as the separation between the locations where the magnetic field amplitude has fallen to 1/*e* of its maximal value in the *z* (*y*) direction, and we denote it by *δ*_*l*_ (*δ*_*t*_). The dependences of ${\bar{H}}_{{\rm{\max }}}$, ${\bar{E}}_{{\rm{\max }}}$, *δ*_*l*_, and *δ*_*t*_ on *ν* are plotted in Fig. 4(a–d), respectively. Note that the hotspot is uniform in the *x* direction. It is found that *δ*_*l*_ = 0.83 mm for *ν* = 10^−4^, *δ*_*l*_ = 0.08 mm for *ν* = 10^−3^, and *δ*_*l*_ = 0.01 mm for *ν* = 0.01, while *δ*_*t*_ is nearly 0.003 mm for all three cases. Similar to *δ*_*t*_, the magnetic-field enhancement ${\bar{H}}_{{\rm{\max }}}$ is weakly affected by *ν*. It is found that ${\bar{H}}_{{\rm{\max }}}$ = 1874 for *ν* = 10^−4^, ${\bar{H}}_{{\rm{\max }}}$ = 1800 for *ν* = 10^−3^, and ${\bar{H}}_{{\rm{\max }}}$ = 1282 for *ν* = 0.01. Evidently, when the material loss becomes serious, the hotspot contracts in the longitudinal direction, thus strong magnetic field at its center is almost preserved. So the hotspot and high magnetic-field enhancement are available even for the cases with serious material losses.

**Figure 5:**
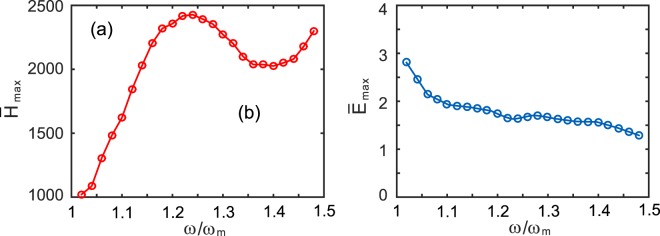
Field amplitude enhancement as a function of frequency for *ν* = 10^−3^. (**a**) ${\bar{H}}_{{\rm{\max }}}$, (**b**) ${\bar{E}}_{{\rm{\max }}}$. The other parameters are the same as in Fig. 1.

In the above analysis, the operation frequency is fixed at *f* = 5.6 GHz, which corresponds to the angular frequency *ω* = 1.12*ω*_*m*_. We further analyze the spectral property of the trapped hotspot. The calculated results are plotted in Fig. 5, where *ν* is set to be 10^−3^. Figure 5(a) shows the value of ${\bar{H}}_{{\rm{\max }}}$ as a function of *ω*. ${\bar{H}}_{{\rm{\max }}}$ reaches a maximum of 2427 at *ω* = 1.24*ω*_*m*_ (i.e., *f* = 6.2 GHz). Figure 5(b) shows the dependence of ${\bar{E}}_{{\rm{\max }}}$ on the frequency. ${\bar{E}}_{{\rm{\max }}}$ slowly decreases with the frequency, and it is less than 3 (but larger than 1) over the whole COWP range [*ω*_*m*_, 1.5*ω*_*m*_].

**Figure 6:**
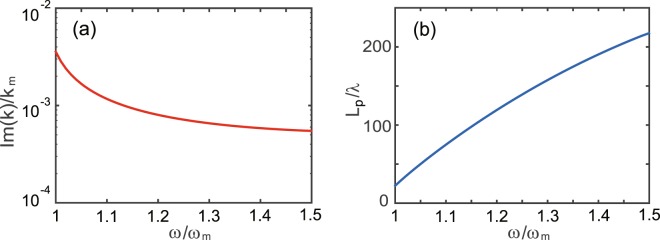
Imaginary part of propagation constant (**a**) and related propagation length (**b**) as a function of frequency for *ν* = 10^−4^. The other parameters are the same as in Fig. 1.

Finally, it is necessary to evaluate the propagation length of the one-way mode in the lossy case. In the lossy case, *k* is a complex number, i.e., *k* = *k*_*r*_ + *ik*_*i*_ (where *k*_*r*_ and *k*_*i*_ are real-valued numbers). Evidently, the influence of *ν* on *k*_*r*_ is negligible for $\nu \ll 1$. Fig. 6(a) shows the value of *k*_*i*_ as a function of frequency for *ν* = 10^−4^. Clearly, *k*_*i*_ decreases with frequency. Note that for a given frequency, *k*_*i*_ is linearly proportional to *ν*. The propagation length, defined by *L*_*p*_ = 1/(2*k*_*i*_), is shown in Fig. 6(b). Evidently, *L*_*p*_ is inversely proportional to *ν*. For example, at *f* = 5.6 GHz, *L*_*p*_ = 83*λ* (i.e., *L*_*p*_ = 4446 mm) for *ν* = 10^−4^ and *L*_*p*_ = 8.3*λ* (i.e., *L*_*p*_ = 444.6 mm) for *ν* = 10^−3^. As the loss of the one-way mode is proportional to the fraction of the EM energy in the YIG region of the waveguide, this energy fraction surely decreases with frequency. From the comparison of Figs 5 and 6, it is clear that the profile of EM energy along *y* for the hotspot should be quite different from that for the one-way mode.

## Pulse Trapping and Compression

In the terminated waveguide system, waves can be completely trapped in it over the whole COWP band [*ω*_*m*_, 1.5*ω*_*m*_]. So it can also completely trap wave pulses as long as their spectra are located within the COWP range. Using the finite-difference time-domain (FDTD) method, we simulated the transmission of a microwave pulse in this system. In the simulation, a Gaussian pulse of linear electric current is employed as source, and its central frequency lies at the center of the COWP band, i.e., *f*_*c*_ = 6.25 GHz. The current source is uniform in the *x* direction, so should be the excited fields in the system. Thus, the FDTD simulation equations in the YIG region can be written as

6 $$\begin{array}{rcl}\frac{\partial {H}_{z}}{\partial y}-\frac{\partial {H}_{y}}{\partial z} & = & {\varepsilon }_{0}{\varepsilon }_{m}\frac{\partial {E}_{x}}{\partial t},\\ \frac{\partial {E}_{x}}{\partial z}-i\frac{\partial {E}_{x}}{\partial y} & = & {\mu }_{0}\frac{\partial {H}_{+}}{\partial t}+{J}_{+},\\ \frac{\partial {E}_{x}}{\partial z}+i\frac{\partial {E}_{x}}{\partial y} & = & {\mu }_{0}\frac{\partial {H}_{-}}{\partial t}+{J}_{-},\\ -{\omega }_{m}(\frac{\partial {E}_{x}}{\partial z}+i\frac{\partial {E}_{x}}{\partial y}) & = & \mathrm{(1}-i\nu )\frac{\partial {J}_{+}}{\partial t}+i({\omega }_{0}+{\omega }_{m}){J}_{+},\\ -{\omega }_{m}(\frac{\partial {E}_{x}}{\partial z}-i\frac{\partial {E}_{x}}{\partial y}) & = & \mathrm{(1}+i\nu )\frac{\partial {J}_{-}}{\partial t}-i({\omega }_{0}+{\omega }_{m}){J}_{-}\mathrm{.}\end{array}$$

where *H*_±_ = *H*_*y*_ ± *iH*_*z*_. In the FDTD simulation, the loss coefficient of YIG is taken to be *ν* = 10^−3^. The length of the waveguide system is 200 mm, and the source is placed at *z* = 50 mm and *y* = 1.5 mm. The linear electric current varied with time as *I*_*m*_ = exp(−(*t* − *t*_0_)^2^/*τ*^2^)exp(−*i*2*πf*_*c*_*t*) (*t* ≥ 0), where *τ* = 1.3*T*_*m*_, *t*_0_ = 4*T*_*m*_ (*T*_*m*_ = 2*π*/*ω*_*m*_), and *f*_*c*_ = 6.25 GHz. The spectral width of this Gaussian pulse, which is defined by Δ*f* = 1/*πτ*, is equal to 1.25 GHz.

**Figure 7:**
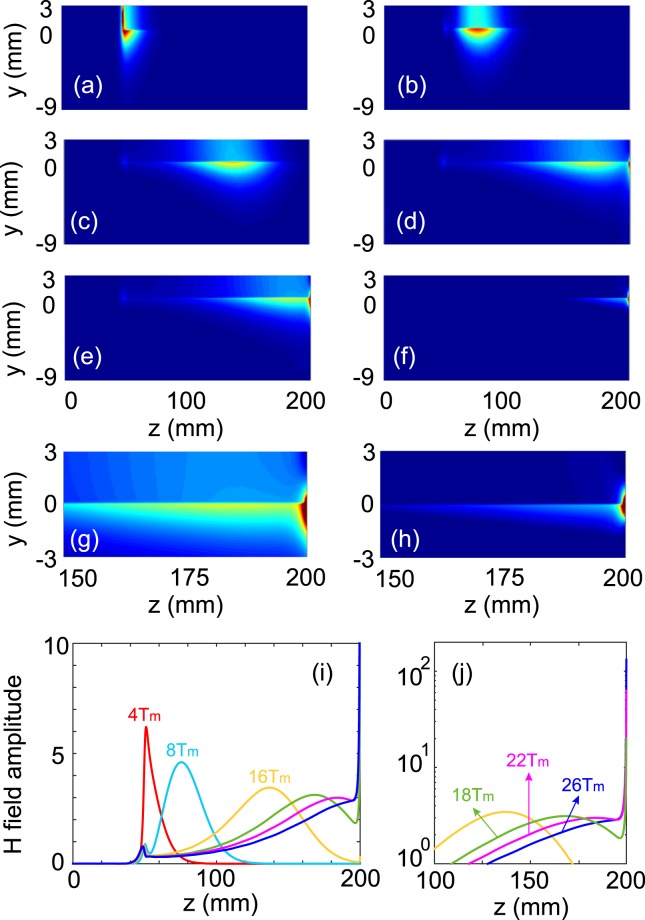
FDTD simulated *H* amplitudes at different evolution times. (**a**) 4*T*_*m*_ (*I*_*m*_ = 1), (**b**) 8*T*_*m*_ (when the excitation finishes), (**c**) 16*T*_*m*_, (**d**) 18*T*_*m*_, (**e**) 22*T*_*m*_, and (**f**) 26*T*_*m*_. (**g**,**h**) Close-up views of local regions in (**e**) and (**f**). (**i**,**j**) Distributions of *H* amplitude on the YIG surface along the *z* axis for the various evolution times. The linear current source is placed at *z* = 50 mm and *y* = 1.5 mm, and its parameters are *τ* = 1.3*T*_*m*_, *t*_0_ = 4*T*_*m*_, and *f*_*c*_ = 6.25 GHz. The loss coefficient is *ν* = 10^−3^ for YIG, and the other parameters are the same as in Fig. 1.

The evolution of simulated *H* field amplitudes with time is displayed in Fig. 7. Figure 7(a) shows the field pattern at *t*_0_ = 4*T*_*m*_, when the Gaussian pulse of the source reached the maximal amplitude, and Fig. 7(b) shows the results at *t*_0_ = 8*T*_*m*_, when the source almost finished the excitation. At this time, an initial wave packet centered at z = 75.5 mm was formed, and its sizes were *δ*_*l*_ = 36 mm and *δ*_*t*_ = 7 mm. As the evolution time went on, the wave packet only traveled forward and simultaneously broadened due to the modal dispersion, as shown in Fig. 7(c) for *t*_0_ = 16*T*_*m*_, at which the wave packet was centered at *z* = 137.5 mm with *δ*_*l*_ = 75 mm. When the wave packet began to touch the terminal end of the system at *z* = 200 mm, it started to be compressed, and meanwhile, the magnetic fields grew rapidly, as shown in Fig. 7(d) for *t*_0_ = 18*T*_*m*_. The wave packet was compressed to about half its longitudinal size at the time *t*_0_ = 22*T*_*m*_, as shown in Fig. 7(e). When *t*_0_ = 26*T*_*m*_, the wave packet was finally compressed to the sizes of *δ*_*l*_ = 2 mm and *δ*_*t*_ = 0.6 mm, and a hotspot with extremely strong *H* field was generated, as shown in Fig. 7(f). Figure 7(g,h) show the close-up views of local regions (near the terminal end) in Fig. 7(e,f), respectively. Figure 7(i,j) show the distributions of *H* field amplitude on the YIG surface along the *z* axis for the different evolution times. The maximal field amplitude of the trapped wave packet in Fig. 7(f) is larger by nearly 21 times than that of the initial wave packet in Fig. 7(b). Hence, by trapping microwave pulse, the wave packet can be compressed to deep subwavelength scale and the magnetic field can be highly enhanced.

## Further Field Enhancement in Tapered Structure

It is clear that the modal property for the one-way waveguide is independent on its lateral width. This offers a possibility to further enhance the magnetic field for trapping wave by tapering laterally the relevant system. To show this, we performed wave transmission in a tapered system, where the lateral width (*w*) of the one-way waveguide is linearly reduced from 5 mm to 1 mm over the waveguide length of 120 mm, i.e., *w* = 5 mm at the open end (*z* = 0) and *w* = 1 mm at the treminal end (*z* = 120 mm). The other parameters of the tapered system are the same as the system with the uniform waveguide in Fig. 3. In the simulation, the open end of the tapered system (*z* = 0) was excited using an input port, as in the case of Fig. 3, and the operating frequency is *f* = 5.6 GHz.

**Figure 8:**
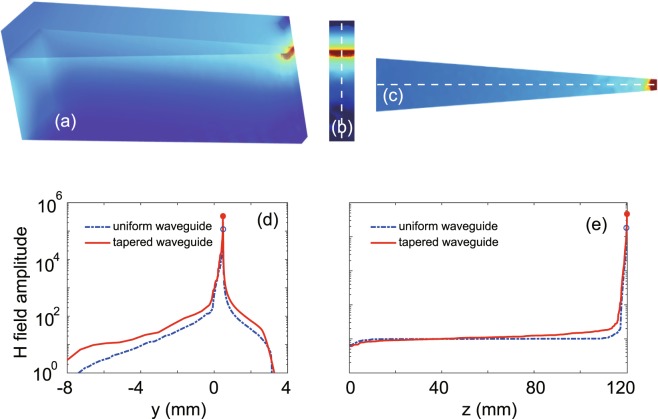
(**a**) Simulated *H* amplitudes in the tapered system for trapping wave. (**b**) Vertical cutting slice in (**a**) at *z* = 119.96 mm, where the center of the hotspot is located. (**c**) Horizontal cutting slice in (**a**) at *y* = 0. (**d**) and (**e**) Distributions of *H* amplitude along the central lines in (**b** and **c**), respectively. The dash-dotted lines in (**d** and **e**) correspond to the results in Fig. 3(b,c) for the system with uniform waveguide. The solid and open circles indicate the peaks of field profiles for the tapered and uniform systems, respectively. In the tapered system, the waveguide width is linearly reduced from 5 mm to 1 mm over the waveguide length of 120 mm. The other parameters are the same as in Fig. 3.

The simulated *H* amplitudes are plotted in Fig. 8(a). Figure 8(b,c) show its vertical cutting slice at *z* = 119.96 mm and the horizontal cutting slice at *y* = 0, respectively. Evidently, incident waves are trapped and a hotspot is generated near the terminal end of the tapered structure, as in the case of Fig. 3, where the one-way waveguide is uniform. But the center of the hotspot lies at 0.04 mm before the terminal end for the present case. Figure 8(d,e) show the distributions of *H* amplitude along the central lines of the slices, which are marked with dashed lines in Fig. 8(b,c). For comparison, the corresponding results in Fig. 3(b,c) for the system with the uniform waveguide are also plotted as dash-dotted lines in Fig. 8(d,e). For the tapered system, the magnetic field enhancement is found to be 3900. In contrast, it is 1800 for the system with the uniform waveguide in Fig. 3. Therefore, in the tapered system, the magnetic-field enhancement is further increased by a factor of 2.17, which is very close to the value of $\sqrt{5}$. Note that the waveguide width at the terminal end in Fig. 8(a) is 5 times smaller than that in Fig. 3(a).

## Conclusion

In summary, a realistic one-way waveguide, which is formed by a rectangular metallic waveguide partly filled with YIG, has been proposed and studied. From the dispersion diagram for this 3D waveguide, one-way mode has been found over a certain frequency range. By the numerical simulation, it has been shown that the one-way mode can be immune to backscattering. A 3D system for trapping microwaves has been further proposed, which is constructed by terminating the one-way waveguide with metal slab. It has been numerically demonstrated that one-way propagating waves can be excited in this system by using an input port, and when incident waves reach the terminal end, they are completely stopped without any backscattering. The trapped waves generate a hotspot with very strong magnetic field. By trapping wave in this manner, the enhancement of the magnetic field amplitude is up to 3 orders of magnitude. Moreover, the material loss only has a weak influence on the magnetic-field enhancement. It has also been demonstrated that for an incident pulse, the wave packet can be completely trapped near the terminal end, and meanwhile, it is compressed to deep subwavelength scale. Furthermore, by tapering laterally the system for trapping wave, the magnetic-field enhancement can be further largely increased. The proposed approach for completely stopping microwaves is feasible in practice and has promising applications in magnetic sensing and magnetic non-linearity.

## References

[1] Stockman MI (2004). Nanofocusing of Optical Energy in Tapered Plasmonic Waveguides. Phys Rev Lett..

[2] Cui Y (2012). Ultrabroadband Light Absorption by a Sawtooth Anisotropic Metamaterial Slab. Nano Letters..

[3] Tonouchi M (2007). Cutting-edge teraherz technology. Nature Photon..

[4] Corcoran B, Monat C, Grillet C (2009). Green light emission in silicon through slow-light enhanced third-harmonic generation in photonic crystal waveguides. Nature Photon..

[5] Gan Q, Fu Z, Ding YJ, Bartoli FJ (2008). Ultrawide-bandwidth slow-light system based on THz plasmonic graded metallic grating structure. Phys Rev Lett..

[6] Tsakmakidis KL, Boardman AD, Hess O (2008). ‘Trapped rainbow’ storage of light in metamaterials. Nature..

[7] Gan Q, Ding YJ, Bartoli FJ (2009). Rainbow trapping and releasing at telecommunication wavelengths. Phys Rev Lett..

[8] He SL, He Y, Jin Y (2014). Revealing the truth about ‘trapped rainbow’ storage of light in metamaterials. Sci Rep..

[9] Yanik MF, Fan S (2009). Stopping light all optically. Phys Rev Lett..

[10] Yanik MF, Suh W, Wang Z, Fan S (2004). Stopping light in a waveguide with an all-optical analog of electromagnetically induced transparency. Phys Rev Lett..

[11] Trabattoni A, Maini L, Benedek G (2012). Stopping light in two dimensional quasicrystalline waveguides. Opt Express..

[12] Shen LF, Zheng XD, Deng XH (2015). Stopping terahertz radiation without backscattering over a broad band. Opt Express..

[13] Shen LF (2015). Complete trapping of electromagnetic radiation using surface magnetoplasmons. Opt Lett..

[14] Yu Z (2009). One-way electromagnetic waveguide formed at the interface between a plasmonic metal under a static magnetic field and a photonic crystal. Phys Rev Lett..

[15] Brion JJ (1972). Theory of Surface Magnetoplasmons in Semiconductors. Phys Rev Lett..

[16] Hu B, Wang QJ, Zhang Y (2012). Broadly tunable one-way terahertz plasmonic waveguide based on nonreciprocal surface magneto plasmons. Opt Lett..

[17] Shen LF (2015). Backscattering-immune one-way surface magnetoplasmons at terahertz frequencies. Opt Express..

[18] Chettiar UK, Davoyan AR, Engheta N (2014). Hotspots from nonreciprocal surface waves. Opt Lett..

[19] Zhang XG, Li W, Jiang XY (2012). Confined one-way mode at magnetic domain wall for broadband high-efficiency one-way waveguide, splitter and bender. Appl Phys Lett..

[20] Hartstein A (1973). Surface polaritons semi-infinite gyromagnetic media. J Phys C..

[21] Deng XH (2015). One-way regular electromagnetic mode immune to backscattering. Appl Opt..

[22] Liu KX, He SL (2016). Truly trapped rainbow by utilizing nonreciprocal waveguides. Sci Rep..

